# A protocol for the development of a validated scale of household water insecurity in the United States: HWISE-USA

**DOI:** 10.1371/journal.pone.0330087

**Published:** 2025-08-11

**Authors:** Amber L. Pearson, Wendy Jepson, Alexandra Brewis, Jeremiah Osborne-Gowey, Amber Wutich, Melissa Beresford, Asher Y. Rosinger, Adam M. Enders, Justin Stoler, the HWISE USA Consortium

**Affiliations:** 1 Charles Stewart Mott Department of Public Health, Michigan State University, Flint, Michigan, United States of America; 2 Department of Geography, Texas A&M University, College Station, Texas, United States of America; 3 School of Human Evolution and Social Change, Arizona State University, Tempe, Arizona, United States of America; 4 Department of Anthropology, San Jose State University, San Jose, California, United States of America; 5 Department of Anthropology, Pennsylvania State University, University Park, Pennsylvania, United States of America; 6 Department of Political Science, University of Louisville, Louisville, Kentucky, United States of America; 7 Department of Geography and Sustainable Development, University of Miami, Coral Gables, Florida, United States of America; SKUMS: Shahrekord University of Medical Science, IRAN, ISLAMIC REPUBLIC OF

## Abstract

**Background:**

New metrics of household water insecurity have been validated for low- to middle-income countries, but it is unclear how these measurements apply to the experiences of people living in high-income countries. This project aims to develop and validate a novel metric for household water insecurity experiences in the United States (HWISE-USA) using a cross-sectional design and data from the Southwest, Midwest, and Western regions.

**Methods:**

We outline the protocol for the development and validation of a novel household water insecurity scale for the United States to address this scientific need, including the following key steps: (1) item development through literature and theory; (2) pre-testing of items and expert review; (3) scale development and item reduction; and (4) scale validation. To assess the performance of the HWISE-USA scale, we will follow the same scale development analytics on a separate, quasi-nationally-representative U.S. sample. The scale will be generated from household survey data collected from communities at risk of water insecurity throughout the United States.

**Discussion:**

We explain how a novel metric of water insecurity experiences for households in the United States has important implications for resource allocation, structural interventions, public health and infrastructure planning, and reductions in inequalities.

**Registration:**

osf.io/zvqs4

## Background

The year 2024 marks the 50^th^ anniversary of the Safe Drinking Water Act (SDWA; 1974) in the United States (U.S.), a feat of engineering, management, public health, and policy covering roughly 148,000 water systems and serving nearly 90% of the U.S. population. Yet, within the U.S., some communities have never experienced the full benefits of such enormous investment in water infrastructure, including rural, migrant, and minoritized communities [1,2]. Others such as Indigenous/tribal communities [3,4] struggle with water insecurity related to colonial dispossession of land and dam development. Further, unincorporated, low-income communities in the U.S. are more likely face systemic exclusion from water development, or ‘underbounding,’ leaving residents to access water from unregulated wells or alternative sources [5–9], and exposing them to higher health risks [10]. Rural, remote, and small communities face regulatory gaps including the lack of requirements for testing and water quality [11]. In addition, households with very low or unpredictable income and/or low quality housing [12] are also at higher risk of water insecurity, defined as a lack of safe, reliable, sufficient, or affordable water for a thriving life [13]. An estimated >1 million people lacked complete plumbing in the U.S. in 2013–2017 [14] with many more low-income households facing unaffordable water rates [15] and at risk of overdue accounts and shutoffs. Even previously reliable systems for providing water to households in the U.S., face emergent challenges. For example, established water systems are increasingly in disrepair [16], have insufficient continued investment in maintenance [17], or rely on lead service lines [18], often leading to tap water mistrust and avoidance [19], and stagnation of water or poor water pressure [20]. Institutional inertia, whereby utility and community water systems focus on pricing, investment, and management at the potential detriment to household water insecurity [12,21]. National trends in drinking water quality violations indicate serious challenges while confirming types of water systems at more risk of a violation. One study reported that in 2015 nearly 21 million people relied on community water systems out of compliance, violating health-based quality standards, with repeat violations higher in rural areas and the U.S. Southwest [22]. These upward trends and vulnerabilities will face increased strain due to climate and hydrological risks [23–25].

In response to various forms of water insecurity and environmental injustices, many low-income residents and communities of color do not trust their tap water and avoid its consumption [19,26–28]. In fact, 61.4 million people in the U.S. do not drink their tap water [29]. A national study found that about 37% of U.S. adults and children reported drinking bottled water on a given day [29], with other studies showing high levels among those with lower levels of education [30], Arizona Latinx adults [31], and minoritized children [32]. The current literature on tap water and bottled water reliance narrowly focuses on mistrust [33,34], neglecting upstream causes of water insecurity such as poor water system management (e.g., Flint, Michigan [35] and Toledo, Ohio [36]), a lack of transparency in billing and fees, water testing only at the treatment facility, and legacies of marginality, unequal governance, and politics embedded in histories of water insecurity [37] and harm [35]. This literature, thus, tends to focus on the importance of aligning water user perceptions with expert technical assessments, and in doing so fails to explain why patterns of mistrust differ over time, and across contexts and populations [35].

## Rationale

This effort to develop a novel water insecurity metric for the U.S. builds on previous work on developing and validating a household water insecurity scale for low- and middle-income countries (LMICs) [38,39]. That project, conducted across 28 sites in 23 countries, created and validated the Household Water Insecurity Experiences (HWISE) scale and subsequently produced a wide range of global health insights. Researchers have demonstrated that household water insecurity is associated with food insecurity, shapes social interactions (e.g., water sharing/borrowing and conflict), influences myriad outcomes including breastfeeding, stress, and psychosocial health [40–43], and is shaped by complex systems of inequality [44]. Yet, there is no equivalent metric validated for high-income countries, as defined by the World Bank for fiscal year 2024 as those with a gross national income (GNI) per capita of $13,846 or more [45], because water insecurity experiences in these settings differ in critical ways [46,47]. Emerging evidence from the U.S. has shown variation in the drivers of perceptions of water insecurity including comparison with neighbors’ water usage, state-level water policy, and the occurrence of droughts in the southeast [48], living conditions in the agricultural water-use sector if the western U.S. [49], a lack of water quality monitoring along the U.S.-Mexico border [50], water testing practices and environmental regulation around hydraulic fracturing in the eastern U.S. [51], and the lack of localized water quality data in the Midwest [52]. The lack of a valid metric, therefore, limits the ability of social and public health scientists to assess and measure water insecurity experiences in these different contexts. While we focus on a U.S.-based scale, it is important to note that household water insecurity is related to aging infrastructure, dispossession, poverty, structural racism, and other linked social, ecological, economic, and political vulnerabilities that have been identified across many other high-income countries including Canada [53,54], Europe [55,56], and Australasia [17,57,58].

## Objective

The primary objective of the current project is to develop and validate a novel metric for household water insecurity experiences in the U.S. (HWISE-USA) using a cross-sectional design across diverse U.S. communities. In doing this, we aim to understand human dimensions of water security at the household level, beyond simply tap water access [13,59]. Given the relatively detailed data available on water systems and water access across the U.S., we focus on a U.S. scale as the first step toward understanding the measurement of water insecurity in high-income contexts.

## Methodology

Here, we outline the protocol for the development and validation of a novel household water insecurity scale for the U.S. to address this scientific need [60]. We follow recommended steps of scale development and validation [60–62], including: (1) item development through literature and theory; (2) pre-testing of items and expert review; (3) scale development and item reduction; and (4) scale validation.

This protocol is organized by steps in the scale development process. First, we describe item development, pre-testing and expert review, and the final candidate scale items. We detail pilot data collection methods, pre-testing of questions, and findings using both target population and expert opinions. Then, we describe survey administration (i.e., sampling, power analysis, and process) followed by analytical stages of scale development, item reduction, and scale validation. We describe tests of dimensionality, reliability, and validity using multiple indicators. The protocol was completed using the SPIROS guidelines [63].

### Ethical approval

Pilot studies were approved by the Michigan State University’s Institutional Review Board (STUDY000002290; date 03/21/2019) and by Arizona State University (STUDY00018008). Informed consent to participate was obtained from all of the participants in the study. In Detroit, consent was obtained in writing. In Phoenix, oral consent was obtained. Consent for publication of the transcripts was not provided.

### Study timeline

Item development, expert review, and pilot testing were all completed in 2023 (see Supplemental File 2 for a timeline). Recruitment for the study outlined in this protocol is ongoing. Recruitment began May 2024 and is anticipated to conclude in August 2025. Data collection is anticipated to conclude August 2025. Results are expected October 2025.

### Item development

Content validity assesses the degree to which scale components represent the construct being studied. Content validity can be assessed through formative research such as literature review, theoretical understandings, pilot studies, and a review of scale content by experts and research participants [61]. In this case, we employed all these strategies to develop the survey items proposed for this study.

### Literature and theory on water insecurity constructs

Based on the authors’ combined experience of over a century of scholarship in water insecurity research, and knowledge from a vast network of researchers [64], we outline six constructs relevant to the measurement of household water insecurity in high-income contexts. In addition, we address severity of water insecurity experience, which further informs our item development process.

1. Affordability.

Affordability refers to the costs required to obtain a necessary level of drinking water service or quantity. Affordability means having adequate and regular access to a vital good or service for residential households. In other words, affordability is not an objective measure [12]. An increasing concern over the affordability of residential drinking water services, particularly for racially minoritized residents in the United States [65], has increased among researchers and policy makers [66–72]. Low-income households with unaffordable water service face welfare-harming choices between water bills and other vital needs. If drinking water is unaffordable, low-income households may reduce water consumption to a level that does not satisfy basic needs. Water shutoffs due to non-payment also cause negative short- and long-term health, household, and economic consequences for affected households [73]. In extreme cases, water shut offs increase risk of child removal from protective services [74]. Concern regarding drinking water affordability in California resulted in implementation of a statewide policy to ensure the human right to safe and affordable water, which is one of the first of its kind in the U.S. [72]. Still, other states lack comprehensive data that quantifies household-level measures of water affordability to examine tradeoffs that people make and how water debt or water shutoffs (or the threat of water shutoffs) may impact peoples’ daily lives, health, and decisions.

2. Water quality.

Water insecurity in the U.S. is often seen as concerns over water quality and organoleptic (i.e., color, taste, and smell) perceptions of tap water [33,34]. In one of the first national studies on tap water perceptions, researchers found that perception of water quality was strongly associated with socioeconomic status and demographics (e.g., education level, income, racial/ethnic minority status, and country of birth), rather than risk factors affecting water safety and quality *per se* [19]. It is important to notes that water quality violations are also more likely to occur in settings with higher concentrations of minoritized populations [75,76]. Reliance on bottled water has also been shown to be higher among those who perceive poor quality as a result of organoleptic properties [30]. Further, small water systems may have regulatory exemption for safe drinking water standards [11,77]. Even among municipal supplies, major water quality failures (e.g., Flint, MI) reported in the media have been shown to affect perceptions of water quality and erode trust in water quality in other areas [78].

3. Emotions.

An increasing body of recent research has highlighted that the difficult lived experience of household water insecurity is distressing and stressful [79,80]. It can trigger strong negative emotions and related stress responses. Particularly, unpredictable, unsafe, and unfairly allocated household water is associated with heightened expressions of distress, frustration, anger, humiliation, despair, worry, distrust, and interpersonal conflict (e.g., [41,81]). These emotional responses around water act as physiological stressors and are generally associated with worse mental health [82–87]. Recent studies have also revealed a correlation between water-related distress and stress-related biophysical changes linked to household responsibility for water management, including elevated blood pressure [88]. Most detailed research demonstrating these associations is from studies in lower-income countries such as Ethiopia and Haiti [89,90]. But there are indications of similar associations in minoritized, water-insecure communities in the U.S. especially in relation to fear and distrust around water quality (e.g., [91,92]).

Research on emotions and water insecurity in high-income countries has focused on acute water crises. For example, research in Flint, Michigan shows how grief, fear, and anger resulting from water insecurity can compound pre-existing challenges such as fatigue, financial insecurity, anxiety, and depression [91,93]. In the case of public water crises, institutional betrayal by governmental officials and a lack of trust and accountability also contribute to increased stress [93], and even predict symptoms of post-traumatic stress disorder [94], persisting for years after major water events [95]. Nascent work has begun to investigate emotions and groundwater security in the U.S. [51], but beyond catastrophic water system failures (e.g., Flint) or disasters (e.g., drought), there are limited studies exploring theorized pathways between chronic or episodic experiences of water insecurity and emotional indicators in high-income countries.

4. Water quantity.

A fundamental dimension of water insecurity is quantity of water, often measured in terms of amount (e.g., gallons per capita per day) available to, collected by, or used by the household, with varying thresholds for exactly what constitutes water adequacy for basic human needs [96]. Yet, such metrics are not comprehensive and may be difficult to answer or irrelevant in high-income contexts. Indeed, water quantity needs will likely vary with the type of premise plumbing available, and bathing, washing, and cooking practices. Since thresholds may not be universal or cross-culturally static, understanding the experience of inadequacy may be more salient, using notions about perceptions about insufficiency or consequences (e.g., thirst).

5. Water reliability.

Reliable water provides communities and households with predictable water supply (i.e., with limited interruptions) to manage daily life [97]. Intermittent water provision (i.e., when water supply is limited for periods of time, supply periods are shortened, or pipes experience regular flow restarting and draining) is a management scheme to address overcapacity that may undermine water quality [98], which is a concern particularly in low- and middle-income settings [99]. In high-income settings, intermittent water may be in the form of water shut-offs [100], a water main break [101] or other infrastructural and system failures (e.g., wells running dry or municipal boil water advisories) [102]. When systems degrade from lack of maintenance, pipes are more prone to breaks [103] and water quality violations [104]. The well-documented decades of disinvestment and political inertia, particularly in the U.S. [105], will only reduce water service reliability into the future, if left unaddressed.

6. Approach coping.

One form of an adaptive coping strategy is approach coping, which is characterized by behavioral activity that is directed towards a stressor or threat such as problem-solving efforts [106]. This contrasts with avoidance coping, whereby the action is directed away from the threat (e.g., denial). Strategies employed to cope with water insecurity have been largely investigated in low-income settings. Most of that work documents behaviors used to mitigate the effects of scarce water, particularly during extended dry periods [107], including migration, limited use of water, change in diet, switch to new water source, and water sharing practices [82,108–110]. Although not investigated on a large scale or in depth, in high-income settings, research suggests that other coping strategies may include shortened or avoidance of showering/bathing [111], reliance on bathing/washing facilities that are not fit for purpose [112], changes in daily life, and changes in eating or cooking practices [113]. This research serves as the first of its kind to examine how frequently households employ each of these coping strategies in high-income settings.

7. Severity.

Most current quantitative assessments of household water insecurity measure the prevalence and frequency of experiences [2,38,59,82,114–116]. While frequency is an accepted measure of exposure or resource insecurity, in general, the *severity* of specific water insecurity dimensions (e.g., affordability, water quality experiences, and shame)—i.e., how intensively they are experienced—has only recently been specified or integrated into research. That is, the inverse correlation of frequency with severity of water insecurity experiences has only been shown in one empirical study to date [79]. Therefore, to advance household water insecurity measurement, theorizations, and subsequently policy interventions, we included questions capturing severity of water insecurity items (i.e., “How disruptive was it in your daily life?”), following Stoler et al. [117]. These questions may be used for weighting items during scale development.

### Pre-testing and expert review

First, a core team of expert researchers with at least 5 years of experience researching household water insecurity and water insecurity scale development co-developed a pilot survey drawing on current literature and formative research [118]. Second, team members conducted sequential waves of pilot survey testing in two sites—Detroit, Michigan (survey only) and Phoenix, Arizona (cognitive interviews and the survey)—to inform revision and refinement of items. Both prior to and after these pilot studies, we convened 35 water scholars and practitioners to engage in expert review to further refine scale item candidate language, cognitive loading, and water insecurity constructs.

### Pilot testing and cognitive interviews

Piloting of water-related survey questions began with a series of items, including the 12 items from the original HWISE scale, with a standard 4-week recall period [39]. Given potential limitations of the relevance of the original HWISE scale in high-income settings [117], we added other items related to affordability, water quality, and approach coping. Thus, our piloted survey included 16 items.

Survey items were pilot tested in English in a sample of adults living across 11 neighborhoods in Detroit, Michigan (May–August 2023), who were enrolled in a longitudinal study [119]. Sampling made use of a cohort sample of residents in high-vacancy, low-income neighborhoods in Detroit, where water shut-offs and other forms of water insecurity have been documented [14,65]. A total of 212 participants took part in the survey. All participants competed the survey; 87% identified as Black or African American and almost 40% earned less than $10,000/year. All participants lived in homes connected to a municipal piped water system. Participants completed the survey in paper form, with staff available in person to assist with completion, reading questions aloud, or any other help requested.

Further refinement began in August 2023 in several waves. First, we conducted 5 intensive cognitive interviews to ensure the survey questions were capturing what was intended. This involved a “think aloud” process with prompting that identified how people were understanding each question and their formulating their answers in relation to the provided items. Then we moved to four rounds of successive piloting and revision of the survey questions, first with undergraduate students, then with targeted purposive sampling with community groups in Maricopa and Pinal County, Arizona, USA (total sample of 304). These pilot interviews were conducted in English by a team of trained fieldworkers, who met at least once weekly to identify any survey issues and adjust the protocol. Targeted purposive sampling (n = 304) was used to capture a maximal range of potential water-insecure households (including peri-urban dependent on wells, lower-income mobile home communities, higher-income suburban without mains, and those living in institutions). Throughout these processes, items that were considered ambiguous or difficult to answer were modified, as recommended [120]. The third and fourth pilot protocol rounds further evaluated timing and participant fatigue, resulting in more condensed survey.

### Expert review

In November 2023 we convened a team of 35 experts from across the U.S. and Europe (all authors) to collaboratively review pilot findings and cognitive interview results, assess survey items, and provide revisions to improve clarity, increase cross-cultural relevance, and reduce burden on the participant. The criteria for selecting experts included: 1) publication within the past five years on water insecurity in a high-income context; or 2) experience developing the low- to middle-income country scale of water insecurity. As a result of this iterative process of survey development, piloting, and expert input, we adopted 19 candidate items for the scale, based on the 16 piloted items, learnings from cognitive interviews, and input from experts.

### Candidate scale items

The survey instrument includes 19 candidate items across six constructs of water insecurity (Table 1) that all ask the respondent to estimate how many times they experienced a given water problem in the prior four weeks. Each item that is affirmed at least once over the prior four weeks is then followed by a question about the severity of the impact of the water insecurity experience (i.e., “How disruptive was it in your daily life?”) with three possible response options: 1 = Not at all; 2 = Somewhat disruptive; and 3 = Very disruptive. These severity scores will be considered for potential weighting of responses in the final scale.

**Table 1:** Candidate items for scale, by water insecurity construct.

	Construct	Question^‡^: “In the last four weeks, how many times…
**1**	Affordability	1. Have you or anyone in your household purchased water (not including your tap water)?”
		2. Have you or anyone in your household had difficulty paying for water?”
**2**	Water quality	3. Did you or anyone in your household experience issues of smell, taste, or color in your tap water?”
		4. Did you or anyone in your household feel concerned about your water quality?”
**3**	Emotions	5. Did you or anyone in your household worry you would not have enough water for all of your household needs?”
		6. Did you or anyone in your household to feel ashamed or embarrassed about your water situation?”
		7. Did you or anyone in your household feel angry about your water situation?”
**4**	Water quantity	8. Have you or anyone in your household gone to sleep thirsty because there was not any water to drink?”
		9. Has there been no useable water whatsoever in your household?
		10. Has there not been as much water to drink as you would like for you or anyone in your household?”
		11. Did you or anyone in your household not have enough safe water necessary to manage health or medical issues?”
		12. Have you or anyone in your household been unable to wash your hands after dirty activities because of issues with water?”
		13. Were you or anyone in your household unable to wash clothes because of problems with water?”
		14. Were you or anyone in your household unable to shower or bathe at home because of your water situation?”
**5**	Water reliability	15. Has your main water source been interrupted or limited?”
**6**	Approach coping	16. Have you or anyone in your household had to change schedules or plans because of problems with your water situation? (Activities that may have been interrupted include caring for others, doing household chores, agricultural work, income-generating activities, and sleeping)”
		17. Have you or anyone in your household had to change what was being eaten because there were problems with water? (Activities that may have been interrupted include caring for others, doing household chores, agricultural work, income-generating activities, and sleeping)”
		18. Did you or anyone in your household have to shower or bathe outside the home because of problems with your water situation?”
		19. Did you or anyone in your household avoid drinking tap water?”

‡ *All responses will be measured continuously.*

Severity for each item also measured, as potential weighting variable, where 1 = Not at all; 2 = Somewhat disruptive; 3 = Very disruptive.

### Planned survey administration

#### Design.

The overall design of the project is a cross-sectional evaluation of water insecurity constructs to create a scale and validate it using multiple measures. A minimum of 1059 participating households from 35–36 sampling clusters from sites in the U.S. will be included in data collection, based on sampling strata and our power analysis (detailed subsequently). The study was registered with OFS [blinded].

### Multistage purposive cluster sampling strategy

Sampling will involve site selection criteria, then cluster selection criteria, and finally random or population-based household selection. All sites are based in the U.S., which is classified as high income according to the World Bank (fiscal year 2024 = $13,846 or more GNI per capita [45]). Sites must also face one of the following water-related challenges: (1) access taken or never provided (e.g., colonial dispossession); (2) degraded infrastructure or poor water management; or (3) climate or hydrological risk. Note that the last category overlaps with the EPA risk assessment items for water systems including, but not limited to: drought events, reliance on snow melt, salt-water intrusion, fire susceptibility, sea level rise, and flooding. Clusters within sites are census units (e.g., tracts, block groups) that must have high proportions of racially minoritized and/or low-income population. Because of recent challenges with study recruitment documented in many settings [121–123], if a site experiences high levels of declined participation, we may expand the cluster definition to neighboring census units. Finally, households will then be sampled using one of two different sampling approaches, based on existing community organizations’ relationships, ongoing research projects, and costs based on collaboration and discussion between the authors and the larger consortium to ensure these decisions are reflective of the protocol. These approaches include random sampling and population sampling (e.g., a census). Inclusion into the study will require site researchers to register their sampling strategy with the study team, provide the census ID for each cluster, and complete a questionnaire on site characteristics. We will collect metadata for site and cluster characteristics including which criteria were satisfied, along with other relevant information including moratoria on water shut-offs, water billing schemes, drought or other weather data, and recent disasters will be compiled at enrollment. We will also compile the census ID information for each cluster. The purpose of the scale to be developed is to generalize to across the U.S. The timing of the surveys will cover all seasons, with the intention of variability across sites (see S1 Fig for timeline).

### Participants

Participants will be adults who reside in each site’s selected clusters. To recruit participants, we will employ a variety of strategies including mailing postcards, conducting recruitment activities (e.g., information booths), via connections to community-based groups, and door-to-door contact with potential participants. We will recruit only one adult (≥ 18y) per household who is familiar with the household’s water situation, which is at the household’s discretion. The reason for this is that research has shown that those with key water responsibilities (e.g., those who ensure that water is available for household members) are adequately familiar with the household water needs and experiences to respond to survey questions [85]. After recruitment and consent, the survey will then be conducted either in-person, by phone, or by video call (e.g., Zoom) using a web application or paper survey for response entry (with field staff available for assistance as needed). All paper surveys will be entered into the online survey database by a trained enumerator.

### Sample size and power analysis

Sample size assessments for the study are based on achieving >80% power to test two hypotheses. We analyzed data collected by the authors from 11 sites in low- and middle-income countries (LMICs) (see Supplementary File 1 for details and the mean and standard deviation values used in calculations) to assist with effect estimates to inform the power calculations.

Our sample size calculations are linked to two tests of predictive validity (see next section). Our hypotheses are: (1) *Households that report bottled water as their primary drinking water source will experience significantly higher water insecurity compared to all other households; and* (2) *Households with higher water insecurity will experience significantly higher perceived stress than households with lower water insecurity.*

For hypothesis 1, we compared mean water insecurity scores (using a scale created for LMICs [39,124]) for those relying on bottled water (bottle or sachet) compared to all other households. For hypothesis 2, we compared mean perceived stress scores [125] between those classed as having higher water insecurity (using the mean as a threshold) versus all other households. For these two hypotheses, we selected the largest required total sample size (for hypothesis 2) of 294. Due to households co-locating within clusters [126], we then increased the target sample size using the variance inflation factor or design effect (DE):


DE = (1+rho*(n−1))\]

where n is the average number of observations per cluster, and rho is the estimated intra-class correlation (ICC).

We assumed 25 households per cluster and an ICC of 0.15. Therefore, we calculated a design effect of 3.6, which was used to increase the sample size to 1059 (=294*3.6 = 1058.4), or 35–36 sampling clusters, each containing 25–30 participants. Note that the target sample here is likely much larger than required for scale development, where the rule of thumb is often cited as 10 participants per scale item [120].

## Statistical plan for scale development and validation

### Item reduction, internal consistency, and factor analysis

We will perform all statistical analyses using R software [127]. As a first step, we will examine the distribution of each of the candidate items, measured continuously. We included continuous responses with the intention of promoting participant accuracy in responses [60], and reducing non-construct variance [128].

Next, we will conduct several tests to obtain an optimal model that represents the scale’s latent constructs. To do this, we will first follow standard item reduction analyses that result in an optimal number of items to represent household water insecurity experiences for high-income countries. We will start with the candidate items (Table 1), then exclude items with >20% missingness. We will then evaluate the correlation matrix of the remaining items (see Table 2). This procedure may also result in item reduction as items with factor loadings <0.30 are generally considered inadequate because they contribute <10% variation of the latent construct measured [129]. We will also conduct the Bartlett test to check correlations between variables, our cutoff at p ≤ 0.05. Another sample adequacy test employed in this analysis will be the Kaiser-Meyer-Olkin (KMO), which is used to assess the factorability of the data [130].

**Table 2:** Type, description, and method to assess reliability and validity.

Type	Description	Method to assess
Content validity	*Degree to which the components of the scale represent the construct being studied*	Literature review, theoretical understandings, pilot studies, review by experts and end-users
Internal consistency	*Degree to which similar items within a scale correlate with one another*	Correlation matrix, Kaiser-Meyer-Olkin test (≤.60), Factor analysis
One-dimensionality	*Degree to which the resulting scale items are one-dimensional*	Unidimensionality Congruence (UniCo), Common Explained Variance (ECV)
Reliability	*Degree to which scale is internally consistent and homogenous*	Cronbach’s α (≥.70)
Model fit		Root Mean Square Error of Approximation (RMSEA <0.08), Comparative Fit Index (CFI, > 0.90), Tucker-Lewis Index (TLI, > 0.90),
Predictive validity	*Degree to which the measure can predict a future outcome*	T-test, multivariable regression modeling using data on bottled water reliance, stress
Convergent validity	*Degree to which two measures of constructs that theoretically should be related are in fact related*	Compare to HWISE scale using interscale correlation
Discriminant validity	*Degree to which latent variable discriminates from other, latent variables that theoretically should not be related to one another*	Compare to nature program viewing using correlation

Once these tests are completed, we will perform a factor analysis. We may also exclude items that are cross-loaded or that do not load uniquely on individual factors. We will use the robust adjustment index based on the mean and variance-adjusted chi-square statistic for data correction, assuming that the data is not normally distributed. We will then run several models to extract and generate the factors. We will also investigate inverse probability weighting [131] to accommodate missing data patterns that are not missing at random. We will consider weighting items based on severity of disruption responses.

### One-dimensionality

We will perform a parallel analysis to indicate whether the resulting scale items are one-dimensional. In addition, we will test dimensionality by calculating the Uni-dimensionality Congruence (UniCo) and Common Explained Variance (ECV) indicators. Finalized items are used to create scale scores for reliability and validity tests. At this point in our analysis, the research team will evaluate calculation of scale scores that use unweighted or weighted procedures.

### Tests of reliability

We will apply several tests to assess scale reliability. We will use Cronbach’s alpha (α), which compares the amount of shared variance among the scale items to the amount of overall variance, to assess the degree of internal consistency of the scale items as at least acceptable (≥0.70) but preferably ≥0.80 [132]. Based on previous experience with water insecurity scale development, we will not evaluate Guttman ordering (i.e., a reproducible hierarchy of item severity across sites) as severity experiences are not well known or established in relation to frequency, as discussed previously. We will also assess composite reliability by determining the reliability of the factorial structure [132].

### Evaluating model fit

We will then test the adequacy of the model using the adjustment indexes (Root Mean Square Error of Approximation [RMSEA]; Comparative Fit Index [CFI]; and Tucker-Lewis Index [TLI]). For inclusion in our model, we will consider RMSEA values less than 0.08, and CFI and TLI values above 0.90, preferably above 0.95 [133–135].

### Tests of predictive validity

#### Bottled water reliance.

A growing body of research demonstrates that low-income and non-White U.S. households disproportionately experience problems with water access, affordability, quality, and service. In response, many low-income households and minoritized communities rely heavily on bottled water as their primary water source. Several regions across the U.S. have high reliance on bottled water, including Appalachia (37%), particularly among populations with lower levels of education and those who perceive the color, taste, and smell to be poor (i.e., organoleptic perceptions) [30], echoing findings from Arizona among Latinx adults [31] and minoritized children [32].

To measure bottled water reliance, we adapted a survey item from the National Health and Examination Survey (NHANES; [136]): “In the last four weeks, what is your primary drinking water source at home?”. Our survey response options reflect high-income settings and include: municipal/utility water piped to home, small bottled water (e.g., 12 oz, 32 oz, and a gallon), rain water, well water, tanker truck delivery, bottled water delivery, vending machine-purchased water (to fill 5- or 20-gallon containers), and trucked water. Responses will be coded to create a binary variable for bottled water reliance (from relevant options in the choice list) and using a similar approach to other research on tap water avoidance [137].

#### Perceived stress*.*

Measures of generalized self-reported stress have also been associated with water insecurity in high-income settings, including Detroit, Michigan [92]. Evidence from Australia also underscores how severe drought impacts common mental health disorders in rural communities, including among Aboriginal people, due to the associated losses of livelihoods and environmental degradation [138,139]. We will use the 4-item Cohen’s Perceived Stress Scale (PSS-4) [140] measured using a Likert-type scale (0 = Never, 4 = Very often). Questions asked in reverse polarity are reverse scored and then all items are summed for total scores from 0 = low to 16 = max stress. PSS-4 has been validated in multiple populations, shown to be correlated with anxiety and depression scales, and has adequate internal consistency and reliability [141]. The scale has also been associated with water insecurity in low- and middle-income sites [41,82,88,142].

#### Tests of predictive validity.

We will determine the construct validity of HWISE-USA scale by testing the two hypotheses that informed our sample size calculation. We will first compare HWISE-USA scores for households reporting reliance on bottled water to all other households using a t-test. Second, using a t-test we will compare PSS-4 scores for households designated as ‘high water insecurity’ (likely above the mean) versus all other households. We will also fit separate multi-level regression models to test for associations between HWISE-USA scores, bottled water reliance, and PSS-4 after adjustment for potential confounders and accounting for the clustered nature of the data.

### Convergent validity

We will perform a test assessing the degree to which HWISE-USA scores concur with the previously developed HWISE scale, as we expect the two scales to be positively correlated. We will evaluate the correlation between these two scales (assuming at least 60% of the variables included are not overlapping) using an interscale correlation test [143].

### Discriminant validity

Discriminant validity tests the extent to which a latent variable (water insecurity) discriminates from other constructs that theoretically should not be related to it, and are not correlated [144]. For discriminant validity, we will use the question “How frequently do you watch or listen to nature programs on the TV or radio?” Response options will be never, rarely, occasionally, or regularly [145]. Indirect nature contact (via TV or radio) is a construct that has been associated with pro-environmental behaviors [145]. Based on both the water insecurity research and nature contact research, there is no theoretical connection between these constructs. While a strong subjective connection with nature is connected to greater general wellbeing and water insecurity is typically associated with poorer mental health, there are not theories and empirical evidence to suggest that these constructs are directly related.

### Validation of scale in a replication dataset

As a final robustness check, the HWISE-USA scale that we generate using the multi-site survey data will be validated against a quasi-nationally-representative survey of U.S. participants that was collected in the spring of 2024. The national survey contains the identical 19 candidate questions deployed in each site and will be used to replicate the factor analysis and reliability and validity tests described above to assess the extent to which the HWISE-USA scale is appropriate for use with the general population.

Our national survey was compiled in Qualtrics and fielded to an online panel administered by Forthright (beforthright.com) from April 30–May 19, 2024. Forthright used respondent quotas to create a sample (n = 2,770) that matched the 2016–2021 American Community Survey 5-Year Estimates on sex, age, race, income, and education with an oversample on non-White race and income below the US median (defined as <$70,000 using our income categories). Because we used quota sampling, there is no response or completion rate to report. The online survey contained two standard attention checks and additional review for participant speeding and response patterns such as straightlining.

## Organization of the project

All site leads will be responsible for community consultation, relevant institutional human subject approvals, and other required local research approvals, all to be collated with study metadata. Consent will be sought orally or in writing, depending on each institution’s IRB requirements. Plans for publication, dissemination, and data deposition throughout the study period will be reviewed by the study team.

We will administer the survey using SurveyCTO version 2.71 [146], a mobile data collection platform that facilitates survey deployment across multiple sites, streamlines workflows across teams, and standardizes metadata and data collection. SurveyCTO also has built-in tools that allow for the project data manager to conduct near real-time data quality control and flag potential issues with questions and responses. SurveyCTO has built-in language translation tools. SurveyCTO will, thus, provide a comprehensive, secure platform for survey administration and data quality monitoring across research sites. Data compilation and processing will be the responsibility of the corresponding author.

Staff from each site will be required to attend a virtual training consisting of reading the survey protocol, outlining survey administration and data collection methods, and procedures for data management. In addition, data sharing and co-authorship protocols will be provided in a study agreement.

## Conclusion

At the time this investigation was initiated, to our knowledge no study has developed and validated a water insecurity metric for the U.S., similar to scales developed for low-income settings. Without such a metric, researchers, practitioners, and water providers are limited in their ability to assess the effectiveness of water interventions in at-risk communities and provide better evidence-informed policies and practices in high-income countries. The HWISE-USA scale will provide a robust tool for identifying variation in community needs and an evidence base for more efficiently deploying resources for infrastructure repair or expansion, expanding water quality testing, or responding to service interruptions related to natural hazards or other disasters. Dissemination of this tool to researchers, policymakers, and practitioners will involve academic journal publications, presentations at academic conferences, stakeholder meetings, and international agencies.

Here, we are focus on water-vulnerable sites in the U.S., justified by the greater detail of existing national data on household water vulnerabilities [1–7,10,12,14–20,22,29]. Because we have not yet carried out the sampling for the survey to be conducted, we cannot provide the demographic and contextual characteristics of that sample. However, we will also attempt to replicate our findings in a nationally-representative sample. In the future, it will be important to expand validation to politically, ecologically, socially, and economically diverse sites in other high-income regions, including Europe, Canada, and Australasia. The HWISE-USA framework and scale lays the groundwork for cross-national comparisons of water insecurity in high-income contexts.

While the data collected will be used to create and validate a novel scale of household water insecurity cross-sectionally, we also plan future research to examine consequences of water insecurity in high-income countries using a longitudinal design to test causality [120]. Further, the temporal stability (e.g., test-retest reliability) will need to be addressed in future research once adequate baseline data can allow better theorization of water security dynamics in these understudied contexts. Still, we note potential recall bias in survey responses related to water insecurity experiences over a four-week period. Future research could usefully assess stability of household reporting over longer (>4 weeks) recall periods, as now completed for the original HWISE scale [147], or the potential differences in experiences and thus the measurement of more chronic water insecurity conditions, as suggested in recent research in Northeast Brazil [99,148].

**Strengths and limitations**

- There is currently no validated metric of household water insecurity for high-income countries.
- We propose a valuable tool for assessing the causes and consequences of water insecurity among households with varying vulnerabilities.
- This proposed study is cross-sectional and will not assess causality.
- Future developments can assess temporal stability of the scale, or validity for capturing water insecurity experiences using survey items with different recall periods.

## Supporting information

S1 File SPIROS checklist. (PDF)

S2 File Power calculation. (DOCX)

S1 Fig Timeline. (TIF)
