# Supplementary material for: A protocol for the development of a validated scale of household water insecurity in the United States: HWISE-USA
Source: PLoS One. 2025 Aug 11;20(8):e0330087. doi: 10.1371/journal.pone.0330087 (PMC12338780; doi:10.1371/journal.pone.0330087)
Supplement: S2 File — (DOCX) [file pone.0330087.s002.docx]

**Supplementary materials**

*Details for power analysis*

We compiled data collected by the authors, from Tanzania (Morogoro), Uganda (Arua), Mexico (Mérida and Torreón), Columbia (Cartegena), Bolivia (San Borja), Ghana (Accra), Malawi (Lilongwe), Guatemala (Acatenango), Tajikistan (Dushanbe), and Nepal (Kathmandu).

1. We calculated means and standard deviations of water insecurity scale scores for those relying on bottled water versus any other source type (means=8.7 versus 6.1, standard deviations=8.2 versus 6.8). This yielded a required sample size of 264.
2. We calculated mean and standard deviations of perceived stress scores (PSS-4) for those with water insecurity scores above and below the mean (means=7.5 versus 6.6, standard deviations=2.6 versus 2.9). This yielded a required sample size of 294.
