# Supplementary figures and images for: A protocol for the development of a validated scale of household water insecurity in the United States: HWISE-USA
Source: PLoS One. 2025 Aug 11;20(8):e0330087. doi: 10.1371/journal.pone.0330087 (PMC12338780; doi:10.1371/journal.pone.0330087)

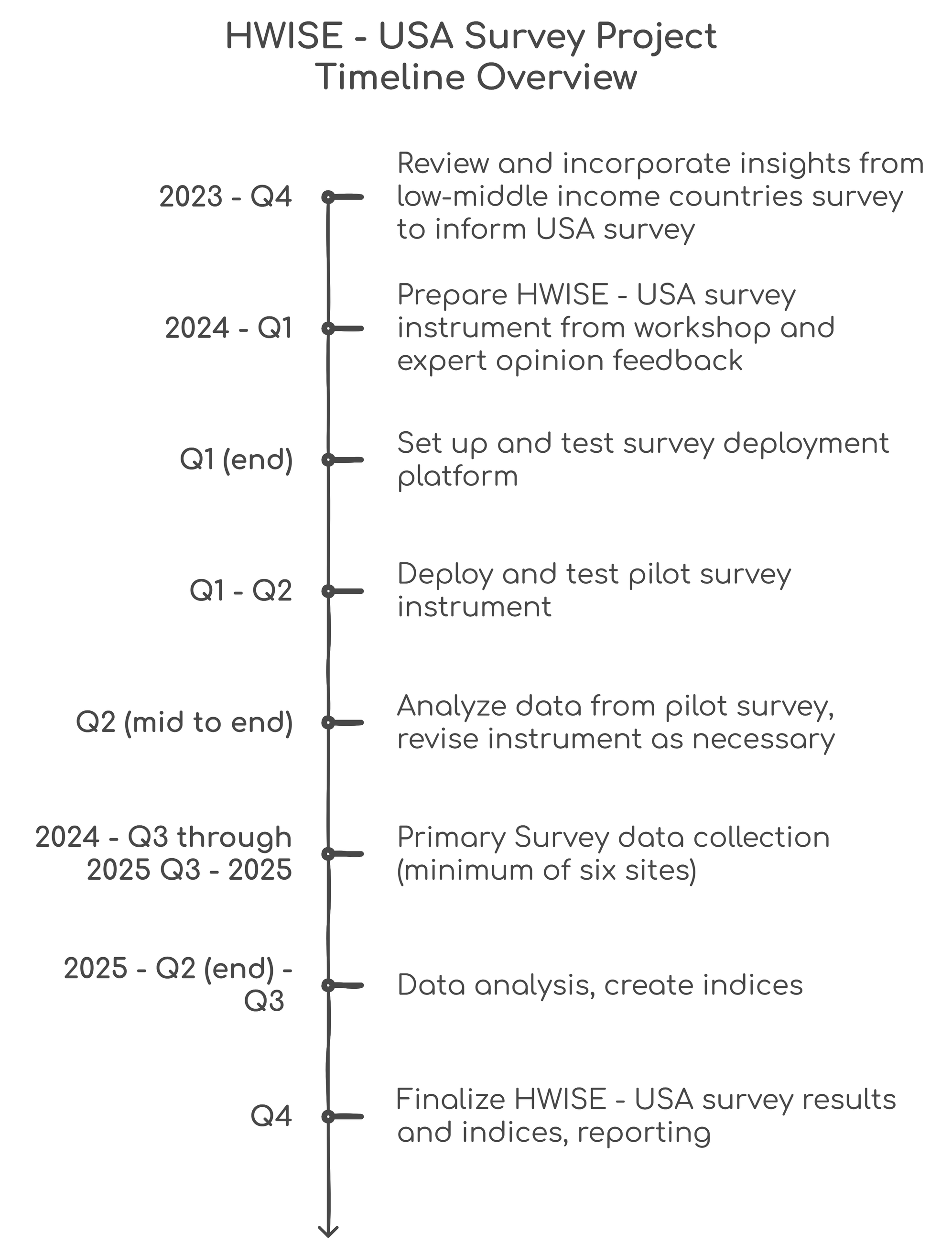

Supplement: S1 Fig — (TIF) [file pone.0330087.s003.tif]
